# Modeling Zika Virus Transmission Dynamics: Parameter Estimates, Disease Characteristics, and Prevention

**DOI:** 10.1038/s41598-019-46218-4

**Published:** 2019-07-22

**Authors:** Munsur Rahman, Kidist Bekele-Maxwell, LeAnna L. Cates, H. T. Banks, Naveen K. Vaidya

**Affiliations:** 1 University of Illinois at Urbana-Champaign, Department of Anthropology, Urbana, 61801 USA; 2 N.C. State University, Center for Research in Scientific Computation, Raleigh, 27695 USA; 3 Department of Global Health and Population, Harvard T.H. Chan School of Public Health, Boston, 02115 USA; 4 San Diego State University, Department of Mathematics and Statistics, San Diego, 92182 USA; 5 San Diego State University, Computational Science Research Center, San Diego, 92182 USA; 6 San Diego State University, Viral Information Institute, San Diego, 92182 USA

**Keywords:** Applied mathematics, Infectious diseases

## Abstract

Because of limited data, much remains uncertain about parameters related to transmission dynamics of Zika virus (ZIKV). Estimating a large number of parameters from the limited information in data may not provide useful knowledge about the ZIKV. Here, we developed a method that utilizes a mathematical model of ZIKV dynamics and the complex-step derivative approximation technique to identify parameters that can be estimated from the available data. Applying our method to epidemic data from the ZIKV outbreaks in French Polynesia and Yap Island, we identified the parameters that can be estimated from these island data. Our results suggest that the parameters that can be estimated from a given data set, as well as the estimated values of those parameters, vary from Island to Island. Our method allowed us to estimate some ZIKV-related parameters with reasonable confidence intervals. We also computed the basic reproduction number to be from 2.03 to 3.20 across islands. Furthermore, using our model, we evaluated potential prevention strategies and found that peak prevalence can be reduced to nearly 10% by reducing mosquito-to-human contact by at least 60% or increasing mosquito death by at least a factor of three of the base case. With these preventions, the final outbreak-size is predicted to be negligible, thereby successfully controlling ZIKV epidemics.

## Introduction

The Zika virus (ZIKV) was first isolated in a Ugandan forest from a febrile rhesus monkey in 1947^1^. The first major outbreaks of ZIKV arose in Yap and Micronesia between April and July of 2007^2^, followed by an additional outbreak in French Polynesia between October 2013 and April 2014^3^. In 2015, ZIKV raised to prominence in American countries, more specifically in Brazil and Colombia^4–6^, the areas where the epidemic form of ZIKV was previously uncommon. In February 2016, the World Health Organization (WHO) declared ZIKV to be a public health emergency of international concern^7^, and the Center for Disease Control (CDC) set their response efforts to a Level 1 activation, which is the highest response level at the agency^8^. This devastating spread of the virus poses a major global public health emergency and prompts worldwide attention.

ZIKV, a member of the Flavivirade family, is primarily vector-borne, with some reported cases of sexual or blood-fusion transmission^1–3,5^. This arbovirus is spread by the *Aedes* genus of mosquito, which is also the primary vector for other well-known viruses like Dengue, Chikungunya, and yellow fever^2,4,6^, and is likely to flourish in tropical areas similar to the French Polynesian landscape. ZIKV symptoms include fever, myalgia/arthralgia, edema of extremities, maculopapular rash, retro-orbital pain, conjunctivitis, and lymphadenopathies^9^, while many ZIKV infected individuals do not show any symptoms at all. Growing evidence shows that ZIKV is linked to several neurological disorders, such as Guillain-Barre Syndrome^10,11^ and microcephaly in infants born to mothers who were infected with ZIKV during pregnancy^12,13^. Unfortunately, there is no specific treatment for this disease, and at this moment the illness cannot be prevented by medications or vaccines. Because of the absence of treatment and vaccines, the immediate control strategy of ZIKV will rely on the control of mosquito and/or human-mosquito contacts. It is thus critical to get insights into the transmission dynamics of ZIKV in the population and properly evaluate potential control strategies.

Mathematical modeling has become a useful tool in studying dynamics and designing prevention and control measures for infectious diseases^14–19^. Previous modeling studies on ZIKV have advanced our understanding of the ZIKV infection and related parameters^2,20,21^, but limited experimental and theoretical studies have left much to be desired. In particular, it has been common practice to estimate the parameters from model fitting without considering that all parameters might not be accurately estimated from the limited data sets^2,21^. Moreover, the number of parameters that can be estimated might not be similar for all datasets. The parameter estimation from model fitting without thorough analysis on the available data set and related estimable parameters may not be reasonable for ZIKV transmission dynamics. Given the lack of detailed analysis on the current parameter estimates, the key epidemiological parameters of ZIKV transmission, including the basic reproduction number, still remain uncertain. In addition, there is a lack of detailed evaluation on potential ZIKV control strategies. Such studies based on prior analysis of parameter estimation and validation could inform future data collection strategy, including those involving prevention measures, such as outbreak planning or assessment of potential countermeasures, thereby helping to decrease the potentiality of this infectious disease to become a pandemic.

The primary objective of this study was to investigate whether data collected from various islands contain information to estimate all the parameters related to ZIKV. For this, we formulated mathematical models of transmission dynamics of ZIKV infection and employed complex-step derivative based sensitivity analysis to identify the parameters that can be estimated from a given limited data. In particular, we used a standard and well-known least square based inverse problem formulation to estimate the parameters. We then performed sensitivity analysis using the relatively unknown and accurate ‘complex-step’ derivative approximation technique to compute sensitivities and standard errors. Using our method, we identified the estimable ZIKV related parameters that can be more confidently estimated from the survey data from six islands of French Polynesia and one island of the Federated States of Micronesia (weekly new infected population). Our techniques allowed us to estimate some ZIKV-related parameters with reasonable confidence intervals. Using these estimated parameters, we computed the basic reproduction number and performed model analysis to study the disease dynamics as well as the effect of prevention programs on disease outcomes.

## Results

### Identification of parameters that can be estimated from island data

We fitted our model to the cumulative new infection data from each of six islands of French Polynesia (Tahiti, Sous-le-vent, Moorea, Tuamotu-Gambier, Marquises, Australes) and Yap island. We first estimated five parameters along with their respective standard errors (Table 1). With these parameters, the model simulations exhibited reasonable agreement with the data (see Supplementary Fig. S1). However, as we can observe from Table 1, the standard errors for the estimated parameters are very large, giving a negative lower limit of 95% confidence intervals. The reason for the large standard errors could be that the data may not have enough information to estimate all five parameters and/or the model solution, *P*, may not be sensitive to all five parameters^22^. This uncertainty embedded in larger confidence intervals can be reduced by using less number of free parameters during data fitting process. As successfully implemented in many previous studies^23–27^, the number of free parameters can be reduced without violating the significance of data-fitting by fixing the parameter which has the least impact on the model solution. To use the similar technique, we computed the sensitivity matrix (see “Methods” section), which allowed us to identify parameters that can be fixed and obtain reasonably smaller confidence intervals without violating the significance of data-fitting.

**Table 1:** Parameters obtained from fitting the model to data with all five parameters estimated.

Parameter	Tahiti	Sous-le-vent	Moorea	Tuamotu-Gambier	Marquises	Australes	Yap	Average of all islands
${\hat{\beta }}_{h}$ *S.Error* [95% CI]	1.5547 0.9818 [−0.4816 3.5910]	0.8794 0.5873 [−0.3387 2.0975]	0.9569 0.5296 [−0.1415 2.0553]	0.9751 2.2552 [−3.7022 5.6524]	0.4601 0.5255 [−0.6298 1.5500]	1.7768 3.4492 [−5.3768 8.9304]	0.1787 3.0457 [−6.1381 6.4955]	0.9688
*β*_*m*_ *S.Error* [95% CI]	0.0561 0.0996 [−0.1505 0.2627]	0.0685 0.0468 [−0.0286 0.1656]	0.1105 0.1887 [−0.2809 0.5019]	0.0771 0.3168 [−0.5799 0.7341]	0.1957 0.4066 [−0.6476 1.0390]	0.0319 0.0943 [−0.1637 0.2275]	1.2873 8.9866 [−17.3509 19.9255]	0.2610
*α*_*h*_ *S.Error* [95% CI]	0.0833 0.0566 [−0.0341 0.2007]	0.1212 0.1431 [−0.0467 0.2133]	0.0875 0.0432 [−0.0021 0.1771]	0.0833 0.2544 [−0.4443 0.6109]	0.2500 0.8910 [−1.0375 1.5375]	0.0833 0.2270 [−0.3875 0.5541]	0.1138 2.8665 [−5.8313 6.0589]	0.1174
*γ*_*h*_ *S.Error* [95% CI]	0.0833 0.2398 [−0.4140 0.5806]	0.0833 0.0627 [−0.0467 0.2133]	0.0833 0.2283 [−0.3902 0.5568]	0.1249 0.5638 [−1.0444 1.2942]	0.2500 0.6208 [−1.0375 1.5375]	0.0878 0.3314 [−0.5995 0.7751]	0.0833 0.7629 [−1.4990 1.6656]	0.1137
η × 100 S.Error [95% CI]	2.8400 0.0142 [2.8105 2.8695]	3.9400 0.0590 [3.8176 4.0624]	2.8500 0.0135 [2.8220 2.8780]	3.9900 0.3638 [3.2355 4.7445]	5.9600 0.5664 [4.7853 7.1347]	11.5800 0.6301 [10.2732 12.8868]	20.0400 4.7154 [10.2603 29.8197]	7.3142

Here, ${\hat{\beta }}_{h}$ and *β*_*m*_ represent mosquito-to-human and human-to-mosquito transmission rate, respectively. Similarly, 1/*α*_*h*_ and 1/*γ*_*h*_ represent human incubation period and the human infectious period. *η* represents proportion of case reported. The average of all islands shown are the values fixed in the subsequent fittings as needed.

**Figure 1:**
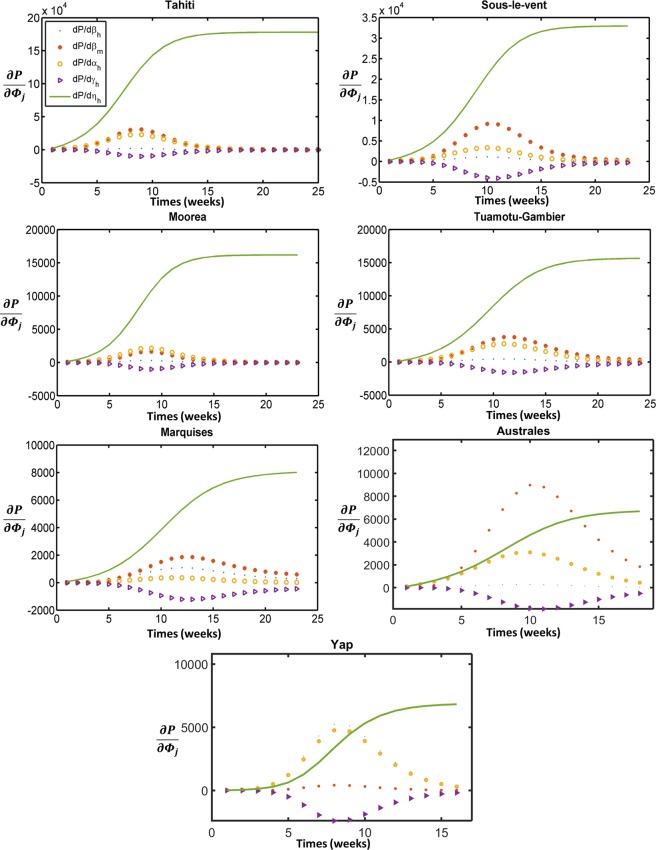
Sensitivity graphs of the cumulative infection *P*. The curves represent the local sensitivity value, $\frac{\partial {\rm{P}}}{\partial {{\rm{\Phi }}}_{j}}$, as a function of time corresponding to ${{\rm{\Phi }}}_{j}={\hat{\beta }}_{h},\,{\beta }_{m},\,{\alpha }_{h},\,{\gamma }_{h},\,{\rm{and}}\,\eta$ at estimated parameter values.

As discussed in “Methods” section, we computed the standard errors and the sensitivity value of *P*, i.e. $\frac{\partial {\rm{P}}}{\partial {{\rm{\Phi }}}_{j}}$, corresponding to all five parameters ${{\rm{\Phi }}}_{j}={\hat{\beta }}_{h},\,{\beta }_{m},\,{\alpha }_{h},\,{\gamma }_{h},\,{\rm{and}}\,\eta$, at the estimated parameter values using the second-order accurate complex-step approximation technique (Fig. 1). Note that the bigger the overall sensitivity value, $\frac{\partial {\rm{P}}}{\partial {{\rm{\Phi }}}_{j}}$, the more sensitive *P* is to the parameter Φ_*j*_. As seen in Fig. 1, the magnitude of the sensitivity of *P* to one of the parameters (mostly *η*) is bigger in a multiple of magnitudes than each of the rest. In addition, the model solution is sensitive to most of the other parameters only for short periods of time. For each island, we identified the least sensitive parameter and fixed it during the data fitting process. We used the fixed value as an average estimate of all islands from Table 1 and later performed the sensitivity analysis of these chosen values. Then we refitted the model to the data to estimate the remaining four parameters (see Supplementary Table S1). We repeated the process by increasing the number of fixed parameters one at a time until each estimated parameter has a confidence interval less than a threshold value (see Supplementary Tables S2 and S3). Since we are interested in 95% confidence intervals, which corresponds to t-value of about 2.1 from student’s t-table for this data, we used a threshold *ϑ* = 1/(2.5) = 0.4 for our estimation (see “Methods” section). However, a lower value can be used if a higher confidence level is desired.

For the data considered here, the standard errors along with sensitivity results suggest that the data sets do not contain sufficient information to estimate more than three parameters in islands of French Polynesia and more than two parameters in Yap island with a reasonable degree of certainty attached to the estimates (Table 2). Interestingly, even in the islands where the equal number of parameters can be estimated, the parameters that can be estimated differ from island to island. For example, the data sets of both Tahiti and S-L-V allow to estimate 3 parameters, but (*β*_*m*_, *α*_*h*_, *η*) can be estimated from Tahiti while (*β*_*m*_, *γ*_*h*_, *η*) can be estimated from S-L-V (Table 2).

**Table 2:** Final parameters estimated with reasonable confidence intervals, estimable parameters, and basic reproduction number (R_0_) with estimated range.

Parameter	Tahiti	S-L-V	Moorea	T-G	Marquises	Australes	Yap
${\hat{\beta }}_{{h}}$ [95% CI]	0.9688 [fixed]	0.9688 [fixed]	0.9688 [fixed]	0.9688 [fixed]	0.9688 [fixed]	0.9688 [fixed]	0.4952 [0.4570 0.5334]
*β*_*m*_ [95% CI]	0.0713 [0.0619 0.0807]	0.0596 [0.0510 0.0682]	0.1325 [0.0888 0.1762]	0.0712 [0.0604 0.0820]	0.0409 [0.0256 0.0562]	0.0536 [0.0475 0.0597]	0.2610 [fixed]
*α*_*h*_ [95% CI]	0.2253 [0.1610 0.2896]	0.1174 [fixed]	0.0836 [0.0589 0.1083]	0.0865 [0.0710 0.1020]	0.1174 [fixed]	0.2500 [0.1826 0.3174]	0.1174 [fixed]
*γ*_*h*_ [95% CI]	0.1137 [fixed]	0.0833 [0.0600 0.1066]	0.1137 [fixed]	0.1137 [fixed]	0.0833 [0.0263 0.1403]	0.1137 [fixed]	0.1137 [fixed]
η × 100 [95% CI]	2.8600 [2.8306 2.8894]	3.9500 [3.9239 3.9761]	2.8500 [2.8275 2.8725]	3.9900 [3.9465 4.0335]	5.7000 [5.4656 5.9344]	11.7800 [11.4940 12.0660]	19.9900 [19.0451 20.9349]
# of estimable parameter	3 (*β*_*m*_, *α*_*h*_, *η*)	3 (*β*_*m*_, *γ*_*h*_, *η*)	3 (*β*_*m*_, *α*_*h*_, *η*)	3 (*β*_*m*_, *α*_*h*_, *η*)	3 (*β*_*m*_, *γ*_*h*_, *η*)	3 (*β*_*m*_, *α*_*h*_, *η*)	2 (${\hat{\beta }}_{{h}}$, *η*)
R_0_ [Range]	2.3383 [2.1787 2.4877]	2.4977 [2.0424 3.1481]	3.1876 [2.6095 3.6759]	2.3367 [2.15222.5076]	2.0691 [1.2613 4.3165]	2.0274 [1.9068 2.1397]	3.1985 [3.0727 3.3196]

### Final parameter estimates

As identified above, the given island data sets allow us to estimate three parameters for Tahiti, S-L-V, Moorea, T-G, Marquises, Australes, and two parameters for Yap with a reasonable confidence interval. Note that the parameters that can be estimated from these data set with a reasonable confidence interval differ from island to island. The final parameters obtained for each island along with their 95% confidence intervals are given in Table 2. The standard errors across all 7 islands have significantly decreased in our final estimates (see Supplementary Fig. S2).

**Figure 2:**
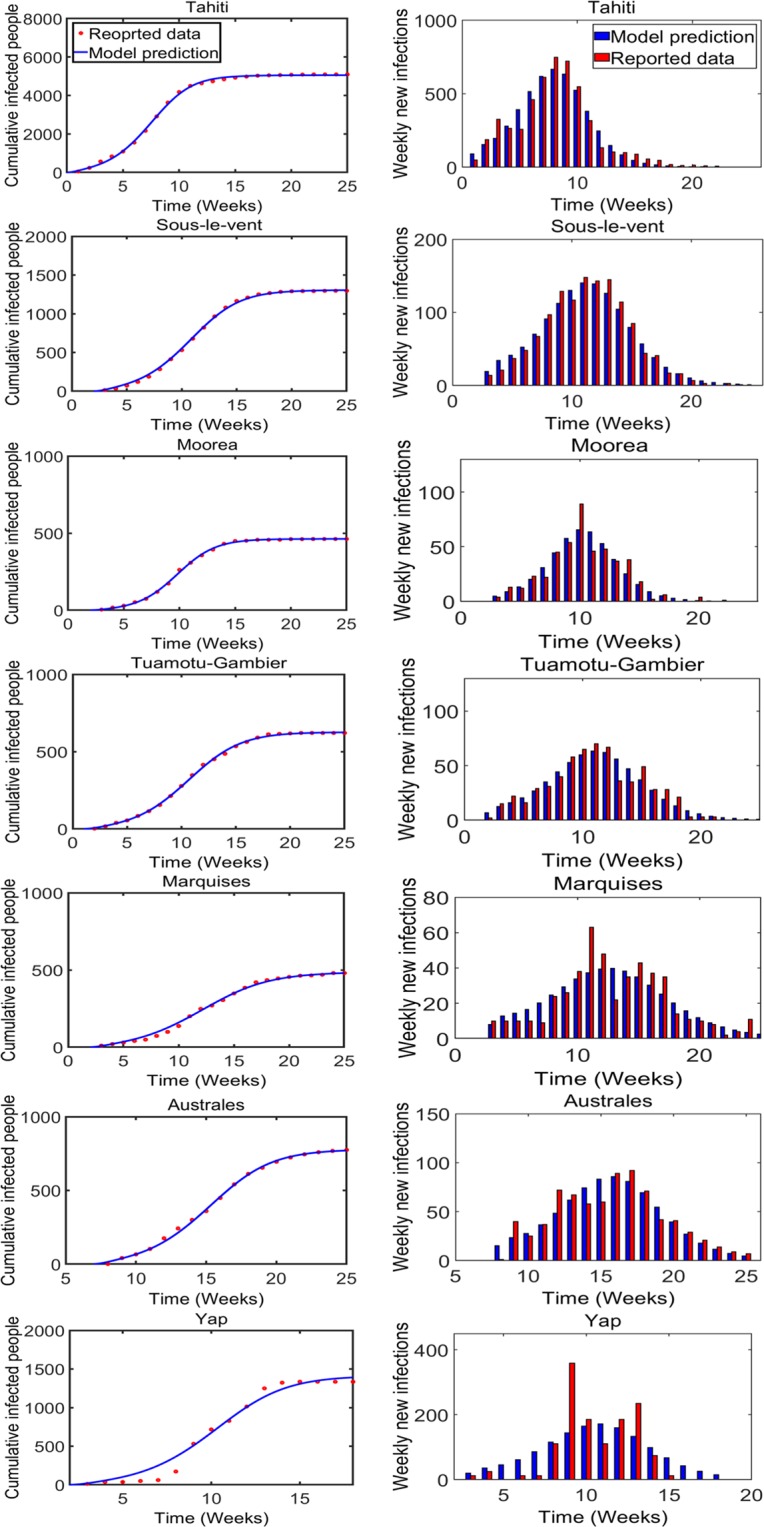
Survey data along with model prediction for each individual island. Cumulative infected humans (left column, solid line: model prediction and dot: data) and weekly new infection (right column, blue: model prediction and red: data).

To assure that the reduction of free parameters does not provide a poor fitting, we performed *F*-test^27^. In each island, we found that increasing the number of free parameters did not improve the statistical significance of the model fitting (p-value > 0.05 in each case). This shows that choosing the fixed parameters in a way as done in our case provides smaller confidence intervals without violating the significance of the data-fitting. With the final estimated parameters, the model prediction along with the survey data for each island is shown in Fig. 2 (left column).

In order to investigate whether the final estimated parameters are affected by the choice of values at which the fixed parameters are set, we performed a sensitivity analysis of the fixed parameters on the estimated parameters. In this analysis, we randomly chose 200 different values for each ‘fixed’ parameter from the uniform distribution of the values over the range of estimate in Table 1. Then for each of 200 sets of fixed parameters, we estimated the free parameters through data fitting. We obtained that the estimated parameters are less sensitive to these fixed values (Supplementary Fig. S3), showing the robustness of our final parameters. This observation is aligned with the fact that fixing the less sensitive parameter at a reasonable value would not significantly affect the estimated parameters.

Note that we estimated parameters based on cumulative data as it provided a simple model formulation. In addition, we also computed the weekly new infection predicted by the model and compared them with the experimental weekly raw data (Fig. 2, right column). The model predictions from these final parameters estimated provide excellent agreement with the experimental weekly data from each of the 7 islands considered. To observe whether these final parameter estimates are affected when weekly raw data are used for fitting as in the early epidemics of Ebola virus^28^, we also fitted our model directly to the weekly raw data and found that the final estimates are not affected much in these island data sets (Supplementary Table S4 and Fig. S4).

### Characteristics of ZIKV transmission dynamics

Note that the mosquito-to-human transmission rate, ${\hat{\beta }}_{h}$, could be estimated with reasonable confidence from only Yap island data. Based on this estimate, we obtained the mosquito-to-human transmission rate to be 0.50 (95% CI: 0.46–0.53) per day for Yap island (Table 2). On the other hand, we could estimate the human-to-mosquito transmission rate, *β*_*m*_, from all islands except Yap, and found that *β*_*m*_ ranges from 0.04 (95% CI: 0.03–0.06) per day in Marquises to 0.13 (95% CI: 0.09–0.18) per day in Moorea. It shows that the per day rate of mosquito-to-human transmission is about 4 to 12 times higher than that of human-to-mosquito. Our predicted human incubation period (1/*α*_*h*_) is about 4 to 12 days and can be estimated from Tahiti, Moorea, T-G and Australes. The predicted infectious (1/*γ*_*h*_) period from our model is about 12 days that was estimated from S-L-V and Marquises islands (Table 2). These predictions are consistent with some previously measured laboratory data^29,30^.

Estimated values of *η*, which could be estimated from the data sets of all islands, indicate that only a small portion of predicted Zika infection was reported to the health sentinel sites. The reported cases ranged from 2.85% in Moorea to 19.99% in Yap. This shows that an actual epidemic size of the ZIKV could be significantly higher than that seemed in the reported cases. This is in agreement with the fact that individuals infected with ZIKV usually do not show any symptoms or show only mild symptoms and are most likely to be unreported.

### Basic reproduction number

The Basic Reproduction Number, *R*_0_, is defined as the average number of secondary cases generated by a typical infectious individual in a fully susceptible population^17^. The disease dies out if *R*_0_ < 1 and the epidemic occurs if *R*_0_ > 1. We calculated *R*_0_ for our model using the next generation operator approach^31^. We obtained the basic reproduction number for our model as follows:

1 $${R}_{0}=\sqrt{\frac{{\hat{\beta }}_{h}\,{\beta }_{m}{\alpha }_{m}}{{\gamma }_{h}{\lambda }_{m}\,({\alpha }_{m}+{\lambda }_{m})\,}}.$$

Using the estimated parameters in Eq. (1), we obtained the basic reproduction number, *R*_0_, with a value ranging from 2.03 in Australes to 3.20 in Yap island (Table 2). Based on the parameter estimates, the range of *R*_0_ for each island is also presented in Table 2. The model predicts *R*_0_ > 1 in each island, and there were ZIKV epidemics, which is consistent with the observations in the data collected.

**Figure 3:**
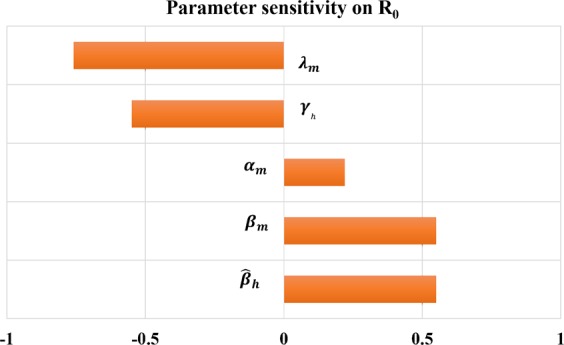
Sensitivity index of the basic reproduction number corresponding to the parameters.

We further examined the effects of the parameters on the reproduction number *R*_0_ using the normalized forward sensitivity index *S*_*x*_ given by^32^:

2 $${S}_{x}=\frac{x}{{R}_{0}}\frac{\partial {{\rm{R}}}_{0}}{\partial {\rm{x}}}$$

where *x* is one of the parameters whose sensitivity on *R*_0_ is sought. This index implies that the higher the value in its magnitude, the more sensitive *R*_0_ is to the parameter. Also, the positive (or negative) sign indicates that *R*_0_ increases (or decreases) as *x* increases. Our result shows that the basic reproduction number is more sensitive to mosquito lifespan than any other parameters (Fig. 3), suggesting that prevention programs focused on reducing mosquito lifespan can be more effective for avoiding ZIKV infection. To a lesser extent, *R*_0_ is also sensitive to *β*_*m*_, ${\hat{\beta }}_{h}$, and *γ*_*h*_. Such measurements can be useful to identify and quantify the effective prevention strategies.

### Disease outcomes: prevalence and outbreak size

**Figure 4:**
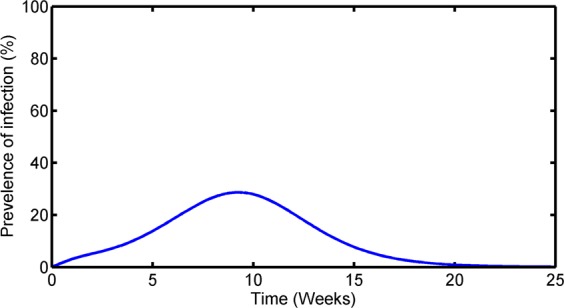
Mean prevalence of infection during the ZIKV epidemic.

While we acknowledge that the same parameters may not be suitable for all the islands, we take the average of the values in Table 2 for our base case computations and simulation study purposes. With these parameters, our model predicts the mean prevalence of infection to be at its peak between the initial 8 to 10 weeks of infection. The amplitude of the peak suggests that during the peak time of infection 30–35% of the total population will be affected (Fig. 4). The model also suggests that after approximately 20 weeks, the ZIKV epidemic will be over, even if no prevention program is implemented. Since our model does not include demographic birth-death and the disease death terms, the final outbreak size can be calculated by integrating the term ${\beta }_{h}{S}_{h}(t){I}_{m}(t)$ from the beginning of infection to the time when epidemic ends. We found that the final size of the epidemic can reach nearly 100% without prevention indicating that almost the entire island population can be infected with ZIKV during the epidemic period.

### Effect of prevention programs on disease outcomes

We evaluated two illustrative prevention programs: one that reduces contact between mosquito and human, and another that decreases the mosquito lifespan. Reducing the contact between mosquito and human refers to a variety of programs, such as wearing skin-covering clothes and using mosquito repellents. Similarly, decreasing the mosquito lifespan refers to the program such as the use of insecticides or other chemicals which aim to inhibit mosquito population growth. In our model, mosquito-to-human (${\hat{\beta }}_{h}$) and human-to-mosquito transmission rate (*β*_*m*_) are the parameters related to prevention programs that focus on reducing contact between humans and mosquitos, while the mosquito life-span (*λ*_*m*_) can be associated with the preventive measures that aim to destruct the mosquito population.

**Figure 5:**
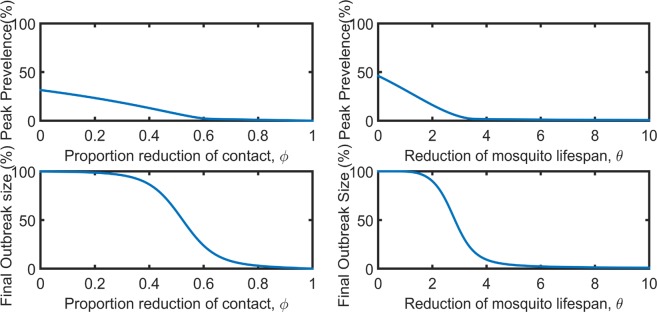
Peak prevalence during an epidemic and final outbreak size predicted by the model for the prevention programs focused on reducing contact between humans and mosquitoes (left column) and mosquito lifespan (right column).

If *ϕ* with 0 ≤ *ϕ* ≤ 1 is an effectiveness of the first prevention program (i.e. the reduction of contact between human and mosquito), implementing such programs causes the following transformation of our model: ${\hat{\beta }}_{h}\to (1-\varphi ){\hat{\beta }}_{h}$ and ${\beta }_{m}\to (1-\varphi ){\beta }_{m}$. Our model suggests that reducing mosquito and human contact by at least 60% (i.e., when *ϕ* ≥ 0.6)would decrease the prevalence of ZIKV to an almost negligible level (Fig. 5). In this case, the final outbreak size reduces dramatically from 100% to nearly 10%.

Similarly, a decrease in mosquito lifespan (the second prevention program) with effectiveness *θ*, i.e., the reduction of mosquito lifespan by *θ* times, changes our model causing ${\lambda }_{m}\to \theta {\lambda }_{m}$. With such prevention programs, the prevalence of ZIKV decreases to a negligible level when mosquito death is increased by at least a factor of three, i.e., *θ* ≥ 3 (Fig. 5). Also, this prevention effort can reduce the final outbreak size from about 100% to nearly 10%.

## Discussion

In this study, we developed a sensitivity analysis based method, which utilizes the transmission dynamics model of ZIKV infection and the recently expanded *complex-step* approximation technique^22,33^, to identify parameters that can be estimated from the available limited data set. Using the estimated parameters by this technique, we also computed the basic reproduction number for ZIKV transmission dynamics and performed analysis and simulation of the models to investigate the disease outcomes and the effectiveness of prevention programs on controlling ZIKV infections.

Implementing our technique to seven island data (six French Polynesia and one Yap), we identified that these data sets do not contain sufficient information to estimate more than three parameters in islands of French Polynesia and more than two parameters in Yap island with a reasonable degree of certainty attached to the estimates. Note that previous studies^21^ used some of these island data to estimate up to six parameters. However, the previous study^21^ used the stochastic approach with a Bayesian fitting procedure and whether this approach experiences similar effects is not known. Importantly, our analysis also showed that the number of estimable parameters and the estimated values varies from island to island, suggesting that the same set of parameters cannot be estimated from every island and thus attempting to estimate the same parameters across all islands may not provide reasonable information about the ZIKV transmission dynamics. Identification of parameters that can be estimated as done in our study may help to obtain important information about parameters related to ZIKV transmission dynamics. As a result, our method provides reasonably small confidence intervals implying more reliability to the estimated parameters (Table 2) while assuring significantly well model fitting to the island data. Compared to a previous study^21^ that used the six islands of French Polynesia, some of the estimates from our method are quite different. In general, our estimates provided higher ${\hat{\beta }}_{h}$, lower *β*_*m*_, and lower *η* than the previous estimates.

We found that only a small portion of infections was reported (2.85–19.99%) as suspected cases across the islands (Table 2). This indicates that actual epidemic size could be quite larger than the documented epidemic size. Those non-reported zika infections might be either asymptomatic infections and/or infections with mild symptoms that did not enter the healthcare system. This phenomenon was supported by the household survey following the Yap island outbreak in 2007^9^. Having a large number of non-reported cases estimated in this study warns higher severity of zika burden in epidemic regions and underscores a need for better surveillance and detection strategies.

Computed basic reproduction number, *R*_0_, from our study slightly varies from island to island (Table 2) and reflects that the ZIKV spreads rapidly throughout the islands. Based on a sensitivity analysis of basic reproduction number, we found that the value of *R*_0_ is mostly dependent on the mosquito life span, though other parameters can also have some impacts on *R*_0_. This indicates that the most effective prevention strategy to avoid zika epidemics could be the control of mosquito growth or life span.

Our investigation on prevalence and infection provided some valuable implications to ZIKV epidemics. The prevalence started to increase at the beginning and reached its peak in between 8 to 10 weeks of the outbreak (Fig. 4). Then, it gradually decreased since more humans were recovering from the virus than those with the new ZIKV infections. Our study found that almost 100% of the island people were infected during the outbreak and the result is consistent with the other studies^21^. Since mosquito bite is the main reason for disease transmission, our result showed that reducing the human and mosquito contact could create a safe environment. Reducing the contact about 60% between human and mosquito can drastically reduce both peak prevalence and final outbreak size and almost eradicate the ZIKV infection (Fig. 5). The outcome is almost identical with the reduction of the mosquito lifespan (Fig. 5). The disease can completely be exterminated by lowering the mosquito lifespan by a factor of 3 to 4 times its base case. We note that the evaluation of these prevention programs was based on the sensitivity of prevention-related model parameters. Further evaluations with detailed models and the data related to the prevention programs are necessary before recommending these programs to practical applications.

We acknowledge some limitations of our study. In this study, we modeled the island situation in which humans and mosquitos usually have close proximity to one another. While our study is relevant to many settings that share characteristics of our population, including military units, college campus, nursing homes, boarding schools, and other rural communities, these results may not be generalizable to other conditions where uniform mixing between humans and mosquitos is not the case. Secondly, we did not consider the seasonal variation in transmission in our analysis as a result of climate factors. However, the outbreaks ended before there was a substantial seasonal change in rainfall or temperature and hence might have very less influence on disease transmission. If the outbreaks had ended because of seasonality rather than the depletion of susceptible populations, it would reduce the estimated proportion of the infected population. Our parameter estimates and related confidence intervals are based on limited data sets, thus there might be some quantitative difference between our predictions and the real scenarios. Our results on prevention programs are based on the parameter sets averaged over islands. However, we acknowledge that the same parameters may not provide reasonable outcomes for all islands, or even for the same island at different time points. We have also ignored potential stochastic effects in ZIKV transmission, which may be important, particularly during the early phase of the infection. The estimates may be improved by incorporating stochastic effects in our model^28^. However, our data contain entire epidemic periods, rather than only initial growth, thereby reducing the stochastic effects. Further study with stochastic modeling is necessary to accurately evaluate the stochastic effects on ZIKV dynamics of these islands.

The main goals of this study were to gain deeper insight into the epidemiological parameters of ZIKV transmission and to evaluate appropriate prevention strategies. The results identified the importance of the information contained in the data in estimating the ZIKV related parameters from the available limited data. This work offered novel insights into ZIKV related parameters as well as ZIKV infection dynamics and effect of prevention programs on disease outcomes, which might be useful for developing ideal prevention and control strategies.

## Methods

### Experimental data

In this study, we utilized the published data containing number of suspected ZIKV infections from six main regions (Tahiti, Iles Sous-le-vent, Moorea, Tuamotu-Gambier, Marquises, and Australes) of French Polynesia, reported weekly between October 2013 and March 2014^34^, and one region of the Federated States of Micronesia (Yap Island), reported weekly between April 2007 and July 2007^9^. In the ZIKV outbreak data of French Polynesia, clinical cases were defined as suspected cases if they were presented to health practitioners with rash and/or mild fever and at least two of the following signs: conjunctivitis, arthralgia, and edema. In total, 8,744 suspected cases were reported from the health sentinel sites. Similarly, in the Yap Island data, researchers reviewed medical records and conducted prospective surveillance at the hospital and all four health centers on Yap to identify patients with suspected ZIKV disease^9^. Suspected cases had the following characteristics: acute onset of generalized macular or papular rash, arthritis or arthralgia, or nonpurulent conjunctivitis. Out of the total 1,276 households tested on Yap Island, 185 cases were identified as suspected ZIKV disease, which we extrapolated for the whole population of Yap Island. We obtained population data for these Islands from the 2012 French Polynesia Census^35^ and the Federated States of Micronesia 2000 Census^36^.

### Mathematical model

**Figure 6:**
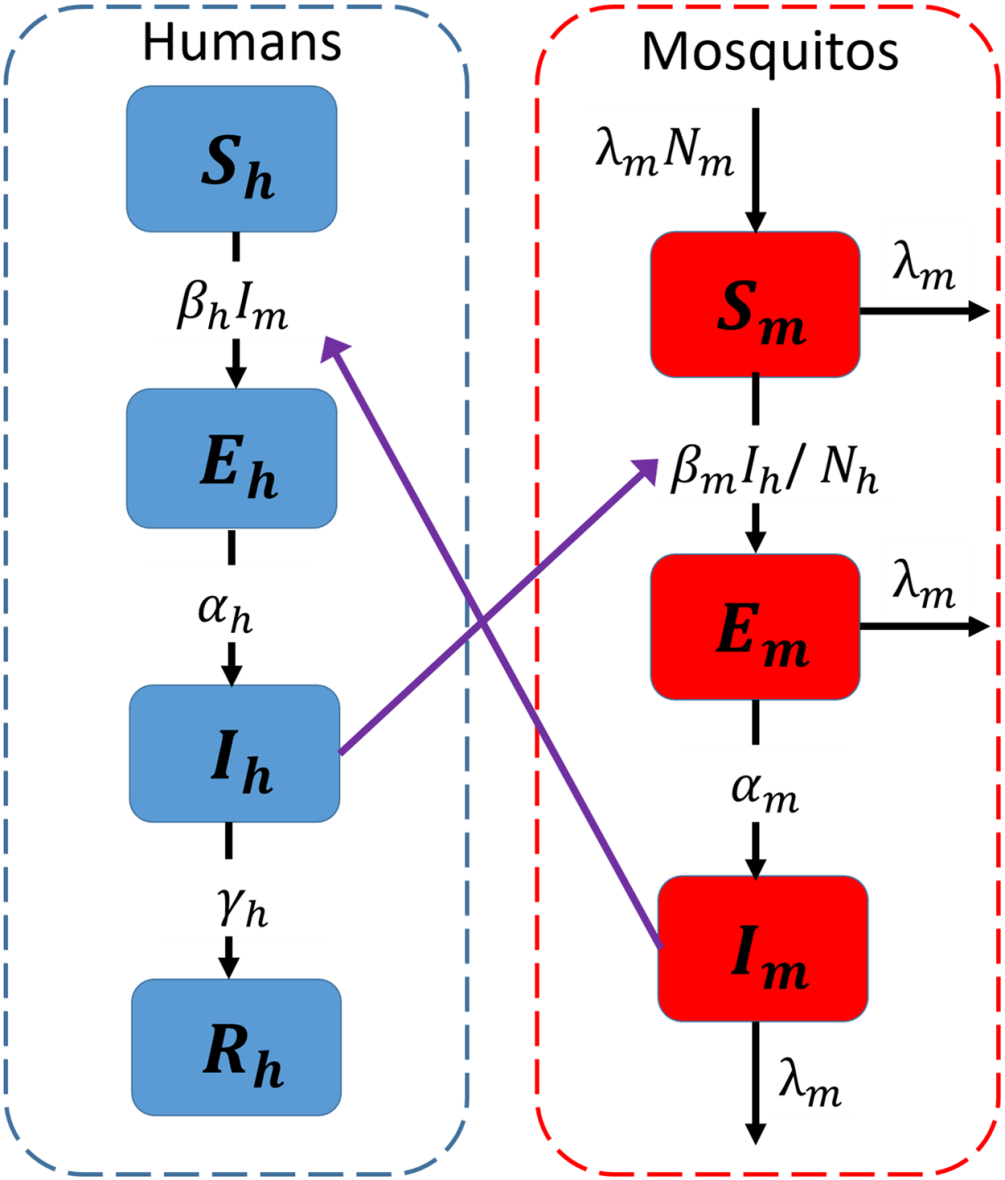
Schematic representation of human-mosquito ZIKV transmission.

We developed a compartmental mathematical model to describe the ZIKV transmission dynamics, similar to the ones previously used for vector-borne transmission^37,38^. The humans were modeled using a susceptible-exposed-infectious-recovered (SEIR) framework, whereas the mosquitos were modeled as susceptible-exposed-infectious (SIE) framework (Fig. 6). In this model, exposed classes were incorporated to include delays as a result of intrinsic (human) and extrinsic (mosquito) incubation periods.

In the model system, *S*_*h*_ represents the number of susceptible humans, *E*_*h*_ is the number of humans currently in their incubation period, *I*_*h*_ is the number of infectious humans, and *R*_*h*_ is the number of humans that have recovered from the ZIKV infection. Similarly, *S*_*m*_, *E*_*m*_, and *I*_*m*_ represent the susceptible, exposed, and infectious mosquito populations, respectively. The dynamics of our ZIKV epidemiological model are governed by the following system:

3 $$\begin{array}{rcl}\frac{d{S}_{h}}{dt} & = & -{\beta }_{h}{S}_{h}{I}_{m}\\ \frac{d{E}_{h}}{dt} & = & {\beta }_{h}{S}_{h}{I}_{m}-{\alpha }_{h}{E}_{h}\\ \frac{d{I}_{h}}{dt} & = & {\alpha }_{h}{E}_{h}-{\gamma }_{h}{I}_{h}\\ \frac{d{R}_{h}}{dt} & = & \,{\gamma }_{h}{I}_{h}\,\end{array}\}$$

4 $$\begin{array}{rcl}\frac{d{S}_{m}}{dt} & = & {\lambda }_{m}{N}_{m}-\frac{\,{\beta }_{m}{S}_{m}{I}_{h}}{{N}_{h}}-{\lambda }_{m}{S}_{m}\\ \frac{d{E}_{m}}{dt} & = & \frac{\,{\beta }_{m}{S}_{m}{I}_{h}}{{N}_{h}}-({\lambda }_{m}+{\alpha }_{m}){E}_{m}\\ \frac{d{I}_{m}}{dt} & = & {\alpha }_{m}{E}_{m}-{\lambda }_{m}{I}_{m}\end{array}\}$$

where ${N}_{h}={S}_{h}+{E}_{h}+{I}_{h}+{R}_{h}$ represents the total number of humans and ${N}_{m}={S}_{m}+{E}_{m}+{I}_{m}$ represents the total number of mosquitos. The parameters 1/*α*_*h*_ represents the human incubation period, 1/*α*_*m*_ is the mosquito incubation period, 1/*γ*_*h*_ represents the human infectious period, and 1/*λ*_*m*_ is the mosquito life-span. In this model, susceptible humans get infected through the bites by infected mosquitos at a mosquito-to-human transmission rate *β*_*h*_ and a susceptible mosquito get infected when it bites infected humans at the human-to-mosquito transmission rate *β*_*m*_. We presented our model with density-dependent infection rate from mosquito to human transmission. In this model, the total human population and the total mosquito population remain constant over time, i.e. $\frac{d{N}_{h}}{dt}=\frac{d{N}_{m}}{dt}=0$. Therefore, with scaling ${\beta }_{h}\to {\beta }_{h}{N}_{h}$, the density-dependent infection rate and the frequency-dependent infection rate are equivalent, and with this scaling, our model can easily recover the model with frequency-dependent rate.

Since the death due to ZIKV was not reported during the period of epidemics, we have ignored disease death rate terms in the model. We also consider a closed population (i.e. a population with no births, deaths or continual immigration), since the mean human lifespan is much longer than the outbreak duration, and entry and exit of people inside the island are negligible during this short period of the outbreak. We assumed all people transmitted at the same rate, regardless of whether they displayed symptoms or were reported as cases. We considered that no transmission typically occurs before the exposed individuals enter the infectious class.

We now introduce variables ${s}_{h}={S}_{h}/{N}_{h}$, ${e}_{h}={E}_{h}/{N}_{h}$, ${i}_{h}={I}_{h}/{N}_{h}$, ${r}_{h}={R}_{h}/{N}_{h}$, ${s}_{m}={S}_{m}/{N}_{m}$, ${e}_{m}={E}_{m}/{N}_{m}$, and ${i}_{m}={I}_{m}/{N}_{m}$, scaled to their corresponding total population size. This allows the standard simplification of ${r}_{h}=1-{s}_{h}-{e}_{h}-{i}_{h}$ and ${s}_{m}=1-{e}_{m}-{i}_{m}$, thereby reducing the population-wide ZIKV model to the following five-dimensional system:

5 $$\begin{array}{rcl}\frac{d{s}_{h}}{dt} & = & -{\hat{\beta }}_{h}{s}_{h}{i}_{m}\\ \frac{d{e}_{h}}{dt} & = & {\hat{\beta }}_{h}\,{s}_{h}{i}_{m}-{\alpha }_{h}{e}_{h}\\ \frac{d{i}_{h}}{dt} & = & {\alpha }_{h}{e}_{h}-{\gamma }_{h}{i}_{h}\\ \frac{d{e}_{m}}{dt} & = & {\beta }_{m}\,{i}_{h}\,(1-{e}_{m}-{i}_{m})-({\lambda }_{m}+{\alpha }_{m}){e}_{m}\\ \frac{d{i}_{m}}{dt} & = & {\alpha }_{m}{e}_{m}-{\lambda }_{m}{i}_{m}\end{array}$$

where $${\hat{\beta }}_{h}={\beta }_{h}{N}_{m}$$.

### Initial population, mosquito lifespan and mosquito incubation period

Serological analysis of samples from blood donors between July 2011 and October 2013 suggested that only 0.8% of the population of French Polynesia were seropositive to ZIKV^39^. We, therefore, assumed that the population was fully susceptible initially. We also assumed that the outbreak began with one initial exposed and one infectious human (i.e., *e*_*h*_*(0)* = *i*_*h*_*(0)* = *1/N*_*H*_), and one exposed and one infectious mosquito (i.e., *e*_*m*_*(0)* = *i*_*m*_*(0)* = *0.005*). The mosquito lifespan and the mosquito incubation period were previously estimated to be 10 days^40,41^ and 15 days^41,42^, respectively. Therefore, we took constant values of 1/*α*_*m*_ = 10 days and 1/*λ*_*m*_ = 15 days for all islands. With these parameters and initial conditions fixed, the remaining five model parameters, *α*_*h*_, *γ*_*h*_, ${\hat{\beta }}_{h}$, *β*_*m*_ and η **(**η is the proportion of case reported) are required to be estimated using epidemic data from ZIKV outbreaks in Yap island and the islands of French Polynesia.

### Model fitting to the data

We fitted the model to cumulative weekly new infection data. The cumulative new infections predicted by our model, *P*(*t*), are given by the solution of the following equation:

6 $$\frac{dP}{dt}={\rm{\eta }}{\alpha }_{h}{e}_{h}{N}_{h}.$$

We solved the system of differential equations numerically using a fourth order Runge–Kutta method. Assuming that the errors are independent and normally distributed with mean zero, we used the solutions to obtain the best-fit parameters via a nonlinear least squares regression method that minimizes the following sum of the squared residuals.

7 $${\rm{J}}({\rm{\Phi }})=\sum _{{\rm{k}}=1}^{{\rm{n}}}\,{[{{\rm{P}}}_{{{\rm{t}}}_{k}}({\rm{\Phi }})-{\bar{{\rm{P}}}}_{{{\rm{t}}}_{k}}]}^{2},$$

where ${\rm{\Phi }}=({{\rm{\Phi }}}_{1},\,{{\rm{\Phi }}}_{2},\,\ldots ,\,{{\rm{\Phi }}}_{m})$ is a set of *m* parameters to be estimated; ${P}_{{t}_{k}}$ and ${\bar{P}}_{{t}_{k}}$ are cumulative infected population values predicted by the model and those obtained from the survey data, respectively. Here, *n* represents the total number of data points available for the model fitting. All computations were carried out in MATLAB (The MathWorks, Inc.). In addition to fitting the model to cumulative data, we also fitted the model directly to weekly new infection data (see “Final parameter estimates” section).

### Computation of confidence intervals

To obtain confidence limits for the estimated parameters, we compute standard errors for Φ by using similar ideas as described in Banks, *et al*.^26^. For this, we first compute the sensitivity matrix Ψ of the parameters.

$$\Psi =[\begin{array}{cccc}\frac{\partial {P}_{{t}_{1}}\,}{\partial {{\Phi }}_{1}} & \frac{\partial {P}_{{t}_{1}}}{\partial {{\Phi }}_{2}} & \ldots  & \frac{\partial {P}_{{t}_{1}}}{\partial {{\Phi }}_{m}}\\ \frac{\partial {P}_{{t}_{2}}\,}{\partial {{\Phi }}_{1}} & \frac{\partial {P}_{{t}_{2}}}{\partial {{\Phi }}_{2}} & \ldots  & \frac{\partial {P}_{{t}_{2}}}{\partial {{\Phi }}_{m}}\\ \vdots  & \vdots  & \ldots  & \vdots \\ \frac{\partial {P}_{{t}_{n}}\,}{\partial {{\Phi }}_{1}} & \frac{\partial {P}_{{t}_{n}}}{\partial {{\Phi }}_{2}} & \ldots  & \frac{\partial {P}_{{t}_{n}}}{\partial {{\Phi }}_{m}}\end{array}].$$

Since we are unable to formulate the closed form of $\frac{\partial {P}_{{t}_{k}}\,}{\partial {\Phi }_{j}}\,,\,j=1,\,2,\,\,\ldots ,\,m,$ and $k=1,\,2,\,\ldots ,\,n$, from our model, we use the following *complex-step* approximation to compute the partial derivatives described briefly below and in Supplementary Materials.

We consider the Taylor expansion of ${P}_{{t}_{n}}$ using a complex step *ih*, where *h* is taken to be a small positive constant (*h* = 10^−40^ in our computations) and *i* is the unit imaginary number.

$${P}_{{t}_{k}}({{\rm{\Phi }}}_{j}+ih)\approx {P}_{{t}_{k}}({{\rm{\Phi }}}_{j})+ih{P}_{{t}_{k}\,}^{\text{'}\text{'}}({{\rm{\Phi }}}_{j})-\frac{{h}^{2}}{2!}{P}_{{t}_{k}\,}^{\text{'}\text{'}}({{\rm{\Phi }}}_{j})+\ldots $$

Taking the imaginary part of both sides of the above equation and dividing by $h$ gives

$${P}_{{t}_{k}}^{^{\prime} }({{\rm{\Phi }}}_{j})=\frac{\partial {P}_{{t}_{k}-}\,}{\partial {\Phi }_{j}}\approx \frac{Im[{P}_{{t}_{k}}({{\rm{\Phi }}}_{j}+ih)]}{h}+O({h}^{2}),$$

where *O*(*h*^2^) represents terms of order 2 and higher. Therefore, the derivatives are given by

$$\frac{\partial {P}_{{t}_{k}}\,}{\partial {\Phi }_{j}}\approx {D}_{h}^{j}({P}_{{t}_{k}})=\frac{Im\,[{P}_{{t}_{k}}({\Phi }_{j}+ih)]}{h},\,j=1,\,2,\,\,\ldots m,\,{\rm{and}}\,k=1,\,2,\,\mathrm{...}\,n.$$

With these, we compute an approximation to the sensitivity matrix *Ψ* denoted by $\hat{\Psi }$. Then we take $\sqrt{{({\sigma }^{2}{\{{\hat{\Psi }}^{T}\hat{\Psi }\}}^{-1})}_{ii}}$, where ${\sigma }^{2}\approx {\hat{\sigma }}^{2}=J({\Phi }^{\ast })/(n-m)$ and Φ* are the basic estimated parameter values, to be the standard deviation for the parameter ${\Phi }_{j},\,j=1,\,2\,\ldots .\,m$. We also compute the standard errors using the usual forward finite difference method for comparison and validation. A brief derivation of the method is provided in Supplementary Materials and more detailed description can be found in Banks, *et al*.^43,44^.

### Sensitivity analysis and stepwise parameter fixation

Since we have available data only on *P*, the information content in the data may not be sufficient to estimate all of the parameters in the model based on the inverse problem formulation. By performing sensitivity analysis, that is, studying the rate of change of *P* due to change in ${{\rm{\Phi }}}_{j},\,j=1\,\ldots ,\,m$, we can determine and identify the maximum number of parameters that can be estimated using each individual island data^26,27^. The procedure of sensitivity driven estimation is as follows.

1. Solve the ZIKV model system with *m* estimated parameters.
2. If the ratio of obtained standard error (*SE*_*j*_) to the estimated parameter value (${{\rm{\Phi }}}_{j}$) is less than a desired threshold (*ϑ*), i.e. $\frac{S{E}_{j}}{{{\rm{\Phi }}}_{j}} < \vartheta$, for each estimate *j* = 1, 2, …, *m*, then STOP. Otherwise, go to step 3 and 4.
3. Choose the parameter which the model solution is least sensitive to (i.e., magnitude of $\partial {\rm{P}}/\partial {{\rm{\Phi }}}_{j}$ close to zero) and fix this parameter at a reasonable value.
4. Estimate remaining *m* − 1 parameters using data-fitting process discussed above.
5. Replace *m* with *m* − 1 and go back to step 1.

Note that the choice of the parameters, which are the least sensitive to the model solution, as done in Step-3 of our algorithm, has been successfully used in many previous studies^23–27^. The rationale for choosing the least sensitive parameters is that it would help maintain the goodness of model fitting to the data, compared to fixing other highly sensitive parameters.

### Statistical analysis

To evaluate the statistical significance of the fits obtained with the model with all five parameters free (Model-a) versus those obtained with the model with some parameter fixed (Model-b), we performed an *F*-test^45^. Note that Model-a and Model-b can be taken as nested models. Similar to Bates and Watts (2007)^45^, we calculate the *F*-ratio = ${s}_{e}^{2}/{s}_{f}^{2}$, where ${s}_{e}^{2}$ is the difference between the residual mean square (RSS) of the two models divided by the number of additional parameters (i.e., two in islands of French Polynesia and three in Yap island). And ${s}_{f}^{2}$ is calculated by dividing the RSS of Model-a by the difference between the number of data points and the number of free parameters. We then compare the ratio with an *F* distribution with the appropriate degrees of freedom.

## Supplementary information

Supplementary Information

## Data Availability

All data generated during this study are included in this published article (and its Supplementary Information files). The analyzed raw data and MATLAB codes are available in the figshare public repository. 10.6084/m9.figshare.5937274.v1, https://figshare.com/articles/Matlab_code_and_data/8148785.
