# Supplementary material for: Modeling Zika Virus Transmission Dynamics: Parameter Estimates, Disease Characteristics, and Prevention
Source: Sci Rep. 2019 Jul 22;9:10575. doi: 10.1038/s41598-019-46218-4 (PMC6646355; doi:10.1038/s41598-019-46218-4)
Supplement: Supplementary file 1 — Supplementary Information [file 41598_2019_46218_MOESM1_ESM.pdf]

Supplementary Information

Modeling Zika Virus Transmission Dynamics: Parameter Estimates, Disease Characteristics, and Prevention

Munsur Rahman[1], Kidist Bekele-Maxwell[2], LeAnna Cates[3], H.T. Banks[2], Naveen K. Vaidya[4,5,6*]

[1] University of Illinois at Urbana-Champaign, Department of Anthropology, Urbana, 61801, USA
[2] N.C. State University, Center for Research in Scientific Computation, Raleigh, 27695, USA
[3] University of Missouri – Kansas City, School of Biological Sciences, Kansas City, 64110, USA
[4] San Diego State University, Department of Mathematics and Statistics, San Diego, 92182, USA
[5] San Diego State University, Computational Science Research Center, San Diego, 92182, USA
[6] San Diego State University, Viral Information Institute, San Diego, 92182, USA

*Corresponding author: nvaidya@sdsu.edu

S1. Complex-step method

Let $z = x + iy, x, y \in \mathbb{R}$ be a complex number and $f(z) = f(x,y) = r(x,y) + iv(x,y)$ be a function of a complex variable. If f is analytic, we have the following Cauchy-Riemann equations that establish the relationship between the real and imaginary parts of the function.

$$\frac{\partial r}{\partial x}(x,y) = \frac{\partial v}{\partial y}(x,y)\ ,\ \ \frac{\partial r}{\partial y}(x,y) = -\frac{\partial v}{\partial x}(x,y). \quad \text{(S-1)}$$

For a given step size h we can derive a finite difference-like first derivative estimate for real functions using complex calculus. From the first equation in (S-1), and the definition of derivatives we have

$$\frac{\partial r}{\partial x}(x,y) = \lim_{h\to 0} \frac{v(x, y+h) - v(x,y)}{h} \quad \text{(S-2)}$$

$$= \lim_{h\to 0} \frac{Im[f(x + i(y+h))] - Im[f(x+iy)]}{h} \quad \text{(S-3)}$$

If f takes a real-valued input, then $y = 0, f(x) = r(x, 0)$, and $v(x, 0) = Im[f(x)] = 0$, Thus (S-2) becomes

$$\frac{\partial f}{\partial x} = \lim_{h \to 0} \frac{Im[f(x + ih)]}{h} \tag{S-4}$$

Therefore, for small h, we have the *complex-step* derivative approximation

$$\frac{\partial f}{\partial x} \approx \frac{Im[f(x + ih)]}{h} \tag{S-5}$$

This formula can also be obtained by approximating a C^2 function *f(q)* with a complex variable using a 2nd order *Taylor* expansion with remainder:

$$f(q + ih) \approx f(q) + ih f'(q) + R(q)$$

where $R(q)$ is $O(h^2)$. Taking the imaginary parts of both sides and dividing by h gives

$$f'(q) \approx \frac{Im[f(q+ih)]}{h} + O(h^2).$$

Terms of order h^2 and higher can be ignored because the step size h can be chosen up to machine precision. Thus, the *complex-step* derivative is given by (S-5) with a truncation error $E_t(h) = \frac{h^2}{6} f^{(3)}(q)$. The method is accurate down to a specific step size we call h_{crit}. Below h_{crit}, underflow occurs, and the approximation becomes useless.

The derivative estimate (S-5) constitutes a big advantage over the finite-difference approach. First, it is applicable for problems with less smoothness than analyticity (e.g., only C^2 functions of the parameters -see [38, 39]). Moreover, the finite-difference approximation is subject to *subtractive error* due to the differencing operation. On the other hand, the accuracy of the complex-step estimates are only limited by the numerical precision of the algorithm that evaluates the function *f*. For a detailed explanation, remarks on implementation procedures, we refer the readers to [38, 39] and the references therein.

S2. Identification of parameters that can be estimated from a limited data

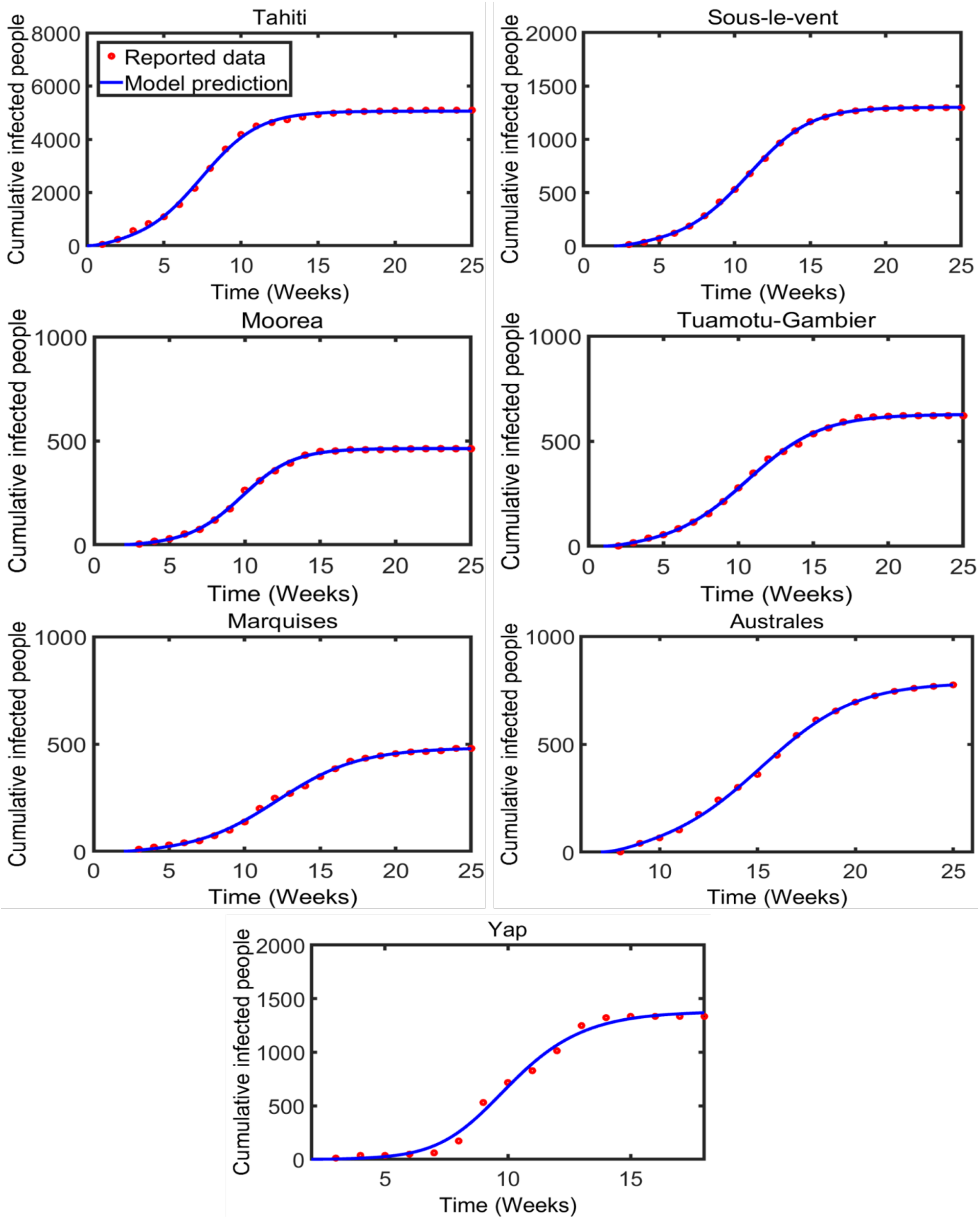

Figure S-1. Results of data fitting for each individual island when all five parameters are estimated. The red circle shows the reported data of the cumulative ZIKV cases, and the solid blue line shows the model predictions.

Table S-1. Estimate of four parameters. Fixed parameters were taken from the average of all island (see Table 1, main text). Based on the error estimates (95% C.I.), none of the island data provided four parameters with reasonable confidence intervals.

Parameter	Tahiti	S-L-V	Moorea	T-G	Marquises	Australes	Yap
$\hat{\beta}_h$	0.9688	0.9688	0.9688	0.9688	0.6596	0.9688	0.4127
S.Error	(fixed)	(fixed)	(fixed)	(fixed)	0.2037	(fixed)	0.7798
β_m	0.0606	0.0676	0.1099	0.0797	0.1591	0.0980	0.2610
S.Error	0.0365	0.0119	0.0341	0.0414	0.3086	0.1133	(fixed)
α_h	0.2500	0.0994	0.0862	0.0833	0.1174	0.2033	0.1433
S.Error	0.1172	0.0087	0.0136	0.0164	(fixed)	0.0951	0.6257
γ_h	0.0910	0.0833	0.0833	0.1293	0.2500	0.2500	0.0833
S.Error	0.0751	0.0185	0.0402	0.0742	0.4718	0.3171	0.0841
η *x 100*	2.8500	3.9300	2.8500	3.9900	5.7700	12.1300	19.8500
S.Error	0.0243	0.0102	0.0130	0.0316	0.2435	0.3281	0.5554

Table S-2. Estimate of three parameters. Fixed parameters were taken from the average of all island (see Table 1, main text). Based on the error estimates (95% C.I.), all data of six islands of French Polynesia provided three parameters with reasonable confidence intervals. Note that the three parameters that can be estimated are not the same for all islands. The Yap island data did not provide three parameters with reasonable confidence intervals.

Parameter	Tahiti	S-L-V	Moorea	T-G	Marquises	Australes	Yap
$\hat{\beta}_h$	0.9688	0.9688	0.9688	0.9688	0.9688	0.9688	0.4590
S.Error	(fixed)	(fixed)	(fixed)	(fixed)	(fixed)	(fixed)	0.8536
β_m	0.0713	0.0596	0.1325	0.0712	0.0409	0.0536	0.2610
S.Error	0.0048	0.0044	0.0223	0.0055	0.0078	0.0031	(fixed)
α_h	0.2253	0.1174	0.0836	0.0865	0.1174	0.2500	0.1377
S.Error	0.0328	(fixed)	0.0126	0.0079	(fixed)	0.0344	0.5635
γ_h	0.1137	0.0833	0.1137	0.1137	0.0833	0.1137	0.1137
S.Error	(fixed)	0.0119	(fixed)	(fixed)	0.0291	(fixed)	(fixed)
η *x 100*	2.8600	3.9500	2.8500	3.9900	5.700	11.7800	19.9600
S.Error	0.0150	0.0133	0.0115	0.0222	0.1196	0.1459	0.4862

Table S-3. Estimate of two parameters in Yap Island. Fixed parameters were taken from the average of all island (see Table 1, main text). Yap island data provided two parameters with reasonable confidence intervals.

Parameter	Tahiti	S-L-V	Moorea	T-G	Marquises	Australes	Yap
$\hat{\beta}_h$<br>*S.Error*	-	-	-	-	-	-	0.4952<br>0.0195
β_m<br>*S.Error*	-	-	-	-	-	-	0.2610<br>(fixed)
α_h<br>*S.Error*	-	-	-	-	-	-	0.1174<br>(fixed)
γ_h<br>*S.Error*	-	-	-	-	-	-	0.1137<br>(fixed)
η *x 100*<br>*S.Error*	-	-	-	-	-	-	19.9900<br>0.4821

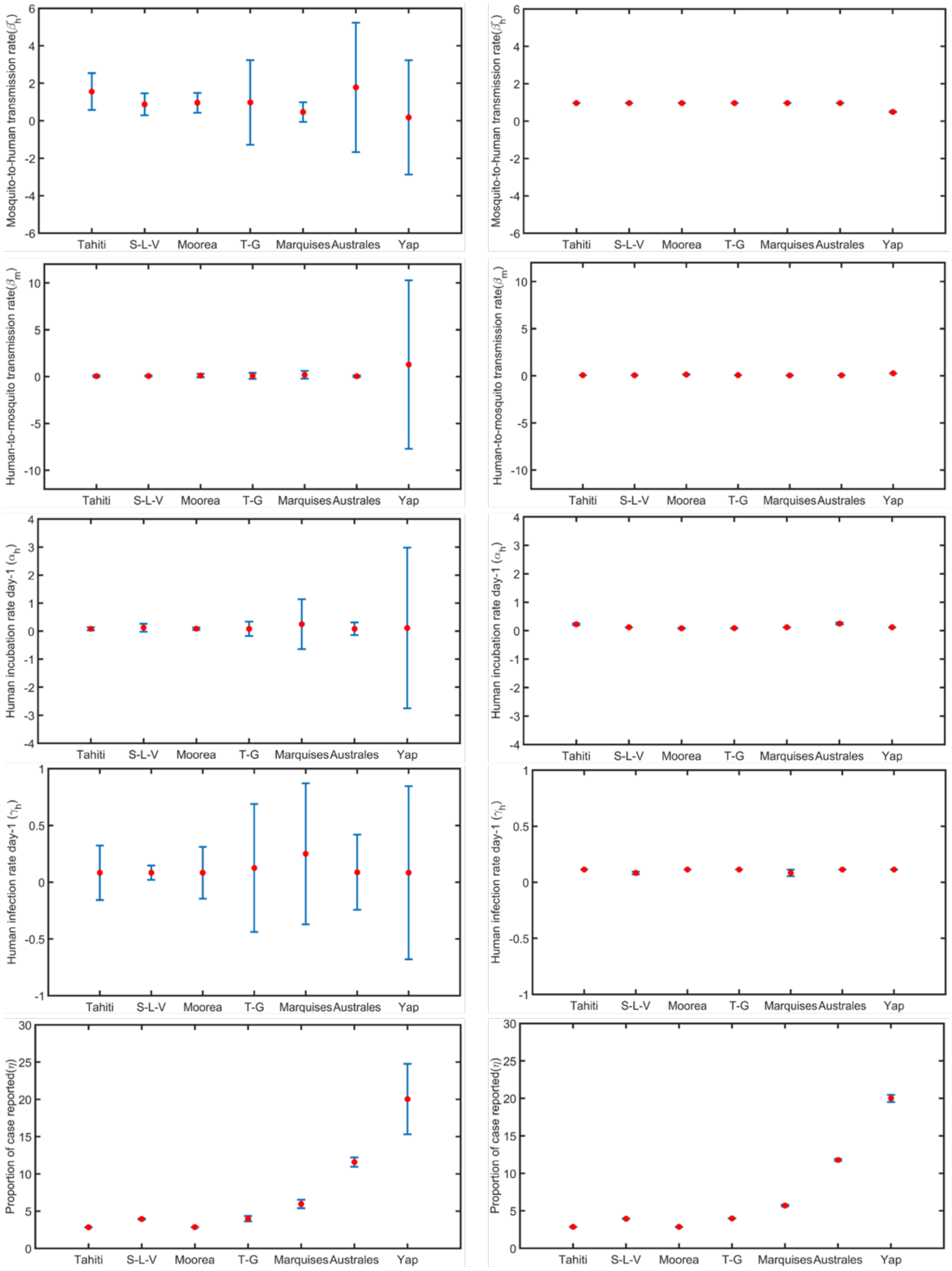

Figure S-2. Estimated parameters (with standard error) obtained from fitting the model when all parameters are estimated (left column) (Table 1, main text) and the final estimates after fixing some parameters (right column) (Table 2, main text).

S3. Sensitivity analysis of fixed parameters on the estimated parameters

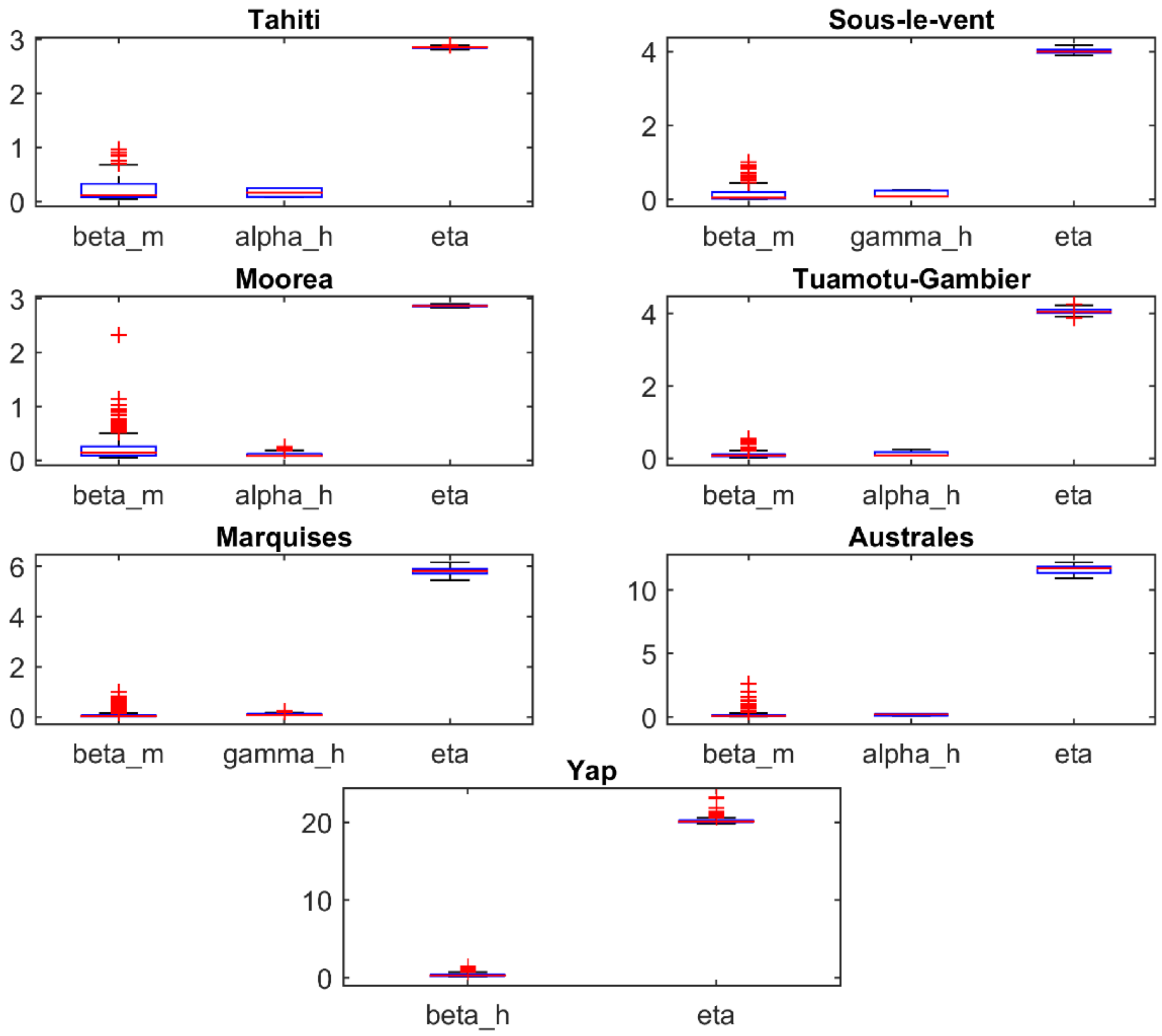

Figure S-3. Sensitivity analysis of the fixed parameters. Box plots showing estimated parameters obtained by choosing 200 random values of fixed parameters from the uniform distribution over the range of values from Table 1 (main text).

S4. Model fitting to weekly new infection (raw) data

Table S-4. The final parameters estimated along with confidence intervals when the model was fitted to the weekly new infection data.

Parameter	Tahiti	S-L-V	Moorea	T-G	Marquises	Australes	Yap
$\hat{\beta}_h$ *[95% CI]*	0.9688 [fixed]	0.9688 [fixed]	0.9688 [fixed]	0.9688 [fixed]	0.9688 [fixed]	0.9688 [fixed]	0.5181 [0.4750 0.5612]
β_m *[95% CI]*	0.0871 [0.0781 0.1160]	0.0594 [0.0517 0.0671]	0.1345 [0.0707 0.1982]	0.0703 [0.0594 0.0811]	0.0424 [0.0278 0.0569]	0.0525 [0.0458 0.0592]	0.2610 (fixed)
α_h *[95% CI]*	0.1357 [0.1005 0.1710]	0.1174 [fixed]	0.0833 [0.0482 0.1184]	0.0889 [0.0724 0.1053]	0.1174 [fixed]	0.2500 [0.1730 0.3270]	0.1174 [fixed]
γ_h *[95% CI]*	0.1137 [fixed]	0.0833 [0.0623 0.1044]	0.1137 [fixed]	0.1137 [fixed]	0.0833 [0.0306 0.1361]	0.1181 [fixed]	0.1137 [fixed]
η *x 100* *[95% CI]*	2.7500 [2.7352 2.7744]	3.9900 [3.9693 4.0172]	2.8600 [2.8282 2.8929]	3.9900 [3.9390 4.0316]	5.7500 [5.5518 5.9487]	11.7800 [11.4683 12.1566]	20.87 [19.8585 21.8873]

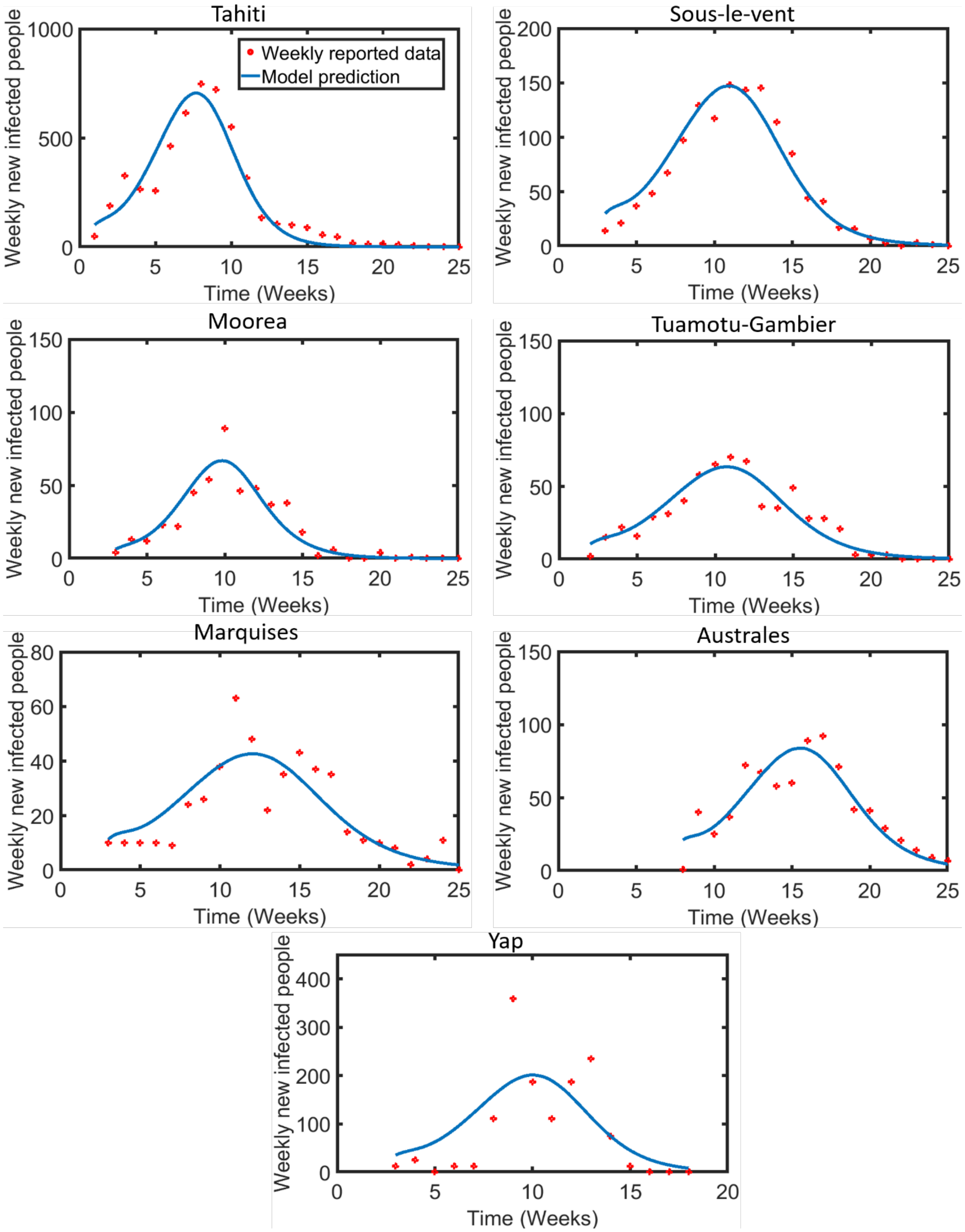

Figure S-4. Survey data along with model prediction for each individual island when the model is fitted to the weekly new infection data. The red dots represent the weekly new infection data and the solid lines represent the model prediction with the parameters given in Table S-4.
